# Supplementary material for: What impact do new homologs have on detecting interdomain horizontal gene transfer in eukaryotes? A reassessment of Katz (2015)
Source: Biol Open. 2026 Mar 25;15(3):bio062387. doi: 10.1242/bio.062387 (PMC13054926; doi:10.1242/bio.062387)
Supplement: Supplementary information [file biolopen-15-062387-s1.pdf]

Table S1. The table reports results obtained using the AVP pipeline for all evaluated candidates. Columns are defined as follows: query name, identifier of the focal eukaryotic sequence; donor, inferred donor lineage; ingroup, taxonomic group containing the query sequence; AI (Alien Index), a score quantifying the relative similarity of the query to non-ingroup versus ingroup homologs; HGT_index, metric reflecting support for horizontal gene transfer based on homology score differences; query hits number, total number of homologous hits retrieved for the query; AHS (Aggregate Hit Support), a contamination-aware score summarizing support for HGT across hits; outg_pct (outgroup percentage), proportion of homologs belonging to outgroup taxa; and Result, final AVP classification for each candidate.

Available for download at
https://journals.biologists.com/bio/article-lookup/doi/10.1242/bio.062387#supplementary-data

Table S2. The table presents the results of the manual phylogenetic evaluation conducted for each candidate. For every analyzed gene, the table records the donor and receptor major clades (MCs), together with a qualitative description of the observed tree topology. The column phylogenetic_pattern_observation categorizes each candidate into recurrent phylogenetic patterns identified in this study, while pattern_description summarizes the main features associated to those phylogenetic pattern. The final conclusion reflects an integrative interpretation of these observations, indicating whether the phylogenetic signal supports horizontal gene transfer (HGT), no HGT or remains inconclusive.

Available for download at
https://journals.biologists.com/bio/article-lookup/doi/10.1242/bio.062387#supplementary-data

Table S3. Presence–absence matrix of homologous sequences for each candidate across major taxonomic groups (MC) and their corresponding minor clades, based on updated homology searches. The table also summarizes changes in major clade representation relative to Katz (2015), including the number of MCs originally reported (Katz_nMC), the number detected in the current analysis (Updated_nMC), the difference between them (deltaMC), and a binary indicator of MC increase (increment_MC): 1=increase, 0=no increase. Increment_MC was used as the response variable in downstream statistical analyses.

Available for download at
https://journals.biologists.com/bio/article-lookup/doi/10.1242/bio.062387#supplementary-data

Table S4. This table summarizes the distribution of HGT candidates across taxonomic groups and compares the original assignments reported by Katz (2015) with those obtained in the present reanalysis. For each taxonomic group, the table reports the major clade (MC) and the corresponding minor clade (mc), together with the number of candidates detected in the original study (candidates_count_Katz) and in the updated analysis (candidates_count_Updated), as well as the associated fold change (FC_candidates_count). The table also includes the taxon sampling used by Katz (n_sp_Katz) and in the current study (n_sp_NR), and the fold change in sampling depth (FC_n_sp). These data form the basis of Figure 2. All MC and mc codes are defined in Table 1 of the article.

Available for download at
https://journals.biologists.com/bio/article-lookup/doi/10.1242/bio.062387#supplementary-data

Biology Open • Supplementary information

Table S5. Query sequences selected for phylogenetic reconstruction and their associated taxonomic lineages.

Available for download at
https://journals.biologists.com/bio/article-lookup/doi/10.1242/bio.062387#supplementary-data

Table S6. Metadata used as input for the AVP analysis, including ingroup definitions, exclusion group parameters (EGP), and their corresponding taxonomic identifiers (taxid).

Available for download at
https://journals.biologists.com/bio/article-lookup/doi/10.1242/bio.062387#supplementary-data
